# Knowledge, Attitude, and Practice of Antibiotic Use and Resistance among Poultry Farmers in Nepal

**DOI:** 10.3390/antibiotics12091369

**Published:** 2023-08-25

**Authors:** Deepak Subedi, Sumit Jyoti, Bhima Thapa, Sanjay Paudel, Prajjwal Shrestha, Deepak Sapkota, Bhuwan Raj Bhatt, Hari Adhikari, Uddab Poudel, Anil Gautam, Rojina Nepal, Ahmad I. Al-Mustapha

**Affiliations:** 1 Department of Poultry Science, University of Georgia, Athens, GA 30602, USA; 2 Paklihawa Campus, Institute of Agriculture and Animal Science, Tribhuvan University, Bhairahawa 32900, Nepal; sujy12@gmail.com (S.J.); sanjay.paudel16@gmail.com (S.P.); prajjwalsth@gmail.com (P.S.); poudeluddab15@gmail.com (U.P.); anilg6623@gmail.com (A.G.); nepalrojina591@gmail.com (R.N.); 3 Department of Health Management, Atlantic Veterinary College, University of Prince Edward Island, 550 University Avenue, Charlottetown, PE C1A 4P3, Canada; 4 Faculty of Animal Science, Veterinary Science and Fisheries, Agriculture and Forestry University, Bharatpur 44200, Nepal; bhimathapa42@gmail.com (B.T.); sapkotadeepak2113@gmail.com (D.S.); drharivetadk@gmail.com (H.A.); 5 Department of Agriculture, University of Arkansas at Pine Bluff, Pine Bluff, AR 71601, USA; 6 Faculty of Agriculture, Far Western University, Kailali 10900, Nepal; bhattabhuwan33@gmail.com; 7 Department of Food Hygiene and Environmental Health, Faculty of Veterinary Medicine, University of Helsinki, 00790 Helsinki, Finland; 8 Department of Veterinary Public Health and Preventive Medicine, Faculty of Veterinary Medicine, University of Ibadan, Ibadan 200284, Nigeria; 9 Department of Veterinary Services, Kwara State Ministry of Agriculture and Rural Development, Ilorin 240213, Nigeria

**Keywords:** antibiotic resistance, AMR, antibiotic use, AMU, KAP, poultry farmers, Nepal

## Abstract

The abuse and misuse of antibiotics is one of the main drivers of antimicrobial resistance (AMR). Globally, AMR in food-producing animals is a significant public health concern. This study, therefore, assessed the knowledge, attitudes, and practices related to antibiotic usage (AMU) and AMR among poultry farmers in Nepal. We conducted a cross-sectional survey of 605 poultry farmers from six districts of Nepal from May to June 2022 to assess the status of knowledge, attitude, as well as practices toward prudent antibiotic usage (AMU) and AMR. The majority of the participants in our study were from the Chitwan district (31.6%; *n* = 191/605), aged 30–44 (54.2%; *n* = 328/605), males (70.4%; *n* = 426/605), and farmers with a higher secondary (28.76%; *n* = 174/605) level of education. The tetracyclines (28%, *n* = 228/828), aminoglycosides (23%, *n* = 188/828), and fluoroquinolones (15%, *n* = 126/828) were the most used antibiotics classes among poultry farmers. Although 87.8% (*n* = 531/605) of poultry farmers used antibiotics, 49.8% (*n* = 301/605) of them were aware of AMR, and 55.7% (*n* = 337/605) knew that the misuse of antimicrobials could affect human and environmental health. There were significant differences in the knowledge, attitude, and practices toward prudent AMU and AMR among farmers who reared different birds. The mean knowledge, attitude, and practice score of the respondents were 7.81 ± 3.26, 5.8 ± 2.32, and 7.59 ± 3.38 when measured on a scale of 12, 10, and 15, respectively. Based on a cut-off of 75% of the maximum score, 49.4% (*n* = 299/605), 62.8% (*n* = 380/605), and 12.73% (*n* = 77/605) of the respondents had good knowledge, attitude, and practices toward prudent AMU and AMR, respectively. The multivariable logistic regression analyses revealed that the positive predictors of good knowledge and attitude were male gender, higher level of education, district, and the types of birds (layers). Similarly, those of the male gender (OR: 3.36; 95% CI: 1.38–8.20; *p* = 0.008) and those that rear layers (OR: 4.63; 95% CI: 1.75–12.25; *p* = 0.003) were more likely to practice prudent usage of antimicrobials. The findings of this study show poor practice toward prudent antibiotic usage despite good knowledge of AMR. This study provides essential baseline data on the knowledge, attitudes, and practices of poultry farmers in Nepal and offers valuable insights that could help in the design of interventions and policies aimed at addressing illicit AMU and AMR in poultry in Nepal.

## 1. Introduction

In resource-limited settings, there is a high usage of antibiotics in food-producing animals for preventive, metaphylactic, therapeutic, and growth promoters, especially with the modernization and commercialization of the poultry industry [1,2,3]. The misuse of antibiotics has been regarded as a major driver of antimicrobial resistance [4]. In addition, it poses a risk of zoonotic transmission as humans may acquire antibiotic-resistant bacteria from animals, particularly poultry, by direct contact with them and their byproducts (meat or eggs) during handling and slaughter, by consuming infected food (farm-to-fork transmission), or by animal waste contaminating the environment [4,5,6,7]. Several studies have provided evidence of the transfer of antibiotic resistance from animals to humans via the food chain [8,9,10].

Nepal, a low-income country in Southeast Asia, has a population of 29.19 million out of which 60.4% of its citizens are engaged in agriculture and livestock production [11]. Agriculture contributes 25.83% of the national gross domestic product (GDP), while the livestock sector alone contributes 13% of the GDP and 27% of the agriculture GDP [11]. In Nepal, poultry farming is rapidly expanding, and the country is self-sufficient in the production of chicken meat and eggs [12]. Around the Kathmandu Valley, there has been a report of high antibiotic usage, including reserve antibiotics for human medicine [13]. Of the total veterinary expenditure in Nepal, antibiotics accounted for 13% in 2008 and this rose to 50% in 2012 [14]. Another study in Nepal revealed that 46% of the veterinary drugs were prescribed by a pharmacist and 12% were due to farmers’ self-demand [2].

Currently, there are no guidelines on prudent antibiotic usage in poultry and food animals in Nepal, there are no laboratory strategic plans for guidance and governance to national laboratories, and most poultry farmers lack adequate training in poultry husbandry, including biosecurity [15]. Hence, proper guidelines on the prudent use of antibiotics in food animals, the establishment of surveillance programs, and biosecurity training programs for livestock farmers, technicians, veterinarians, and the general public on the rational use of antimicrobial drugs can reduce the burden of AMR in Nepal [2]. Baseline data on the knowledge, attitude, and practice of antibiotic use and AMR can play an important role in the design of these training programs that should focus on the rational use of antibiotics, good management practices, and the production of wholesome poultry products [16,17,18]. Therefore, this study evaluated the knowledge, attitude, and practices regarding antibiotic use and resistance among poultry farmers in different regions of Nepal.

## 2. Results

### 2.1. Sociodemographic Characteristics of the Study Population

In our study, a total of 605 responses from individuals from six districts were recorded. The majority of the respondents were from the age group 30–44 (54.21%; *n* = 325/605). Out of the total population, 70.41% (*n* = 426/605) were males and 29.59% (*n* = 179/605) were females. Almost 10% of the total study population had no formal education, and others had other forms of education ranging from primary level to tertiary education (above class 12). Chitwan district had the highest number of participants (31.57%; 191/605), and Syangja district had the lowest participation number (8.93%; *n* = 54/605). More than half of the population in our study were rearing broiler chickens (52.07%; *n* = 315/605). The experience of poultry farming among the study population ranged from less than a year to 40 years. The average (mean ± SD) knowledge, attitude, and practice score of the respondents were 65.1% (7.81± 3.26), 72.5% (5.8 ± 2.32), and 50.6% (7.59 ± 3.38), respectively.

### 2.2. Antibiotics Usage among Poultry Farmers in Nepal

**Figure 1:**
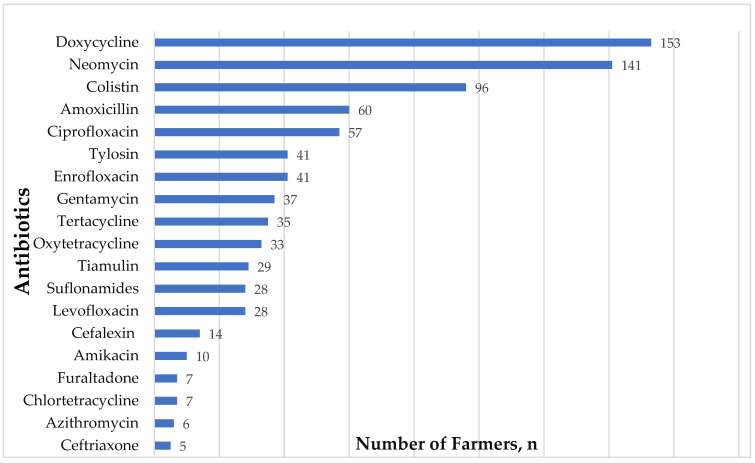
Antibiotics used by poultry farmers in Nepal (*n* = 605).

Out of 605 farmers in our study, 340 farmers could recall 828 different events that prompted them to use antibiotics on their birds. In general, the tetracycline (28%, *n* = 228/828) class of antibiotics was most used by the farmers, followed by aminoglycoside (23%) and fluoroquinolones (15%) (Figure S1). Individually, doxycycline (*n* = 153/828) was the most used antibiotic, followed by neomycin (*n* = 141/828), colistin (*n* = 96/828), and amoxicillin (*n* = 60/828), respectively (Figure 1). Our data also revealed that cephalosporins were not routinely used among poultry farmers in Nepal. Cefalexin (a first-generation cephalosporin) was used by only 14 farmers whereas ceftriaxone (a third-generation cephalosporin) was used by only five of the 605 poultry farmers. Although furazolidone and other members of the nitrofuran class of antibiotics were banned across several regions and countries for their potential carcinogenic effect, seven farmers reported having used the antibiotics in their birds as they are widely available over the counter in Nepal. Additionally, out of 605 farmers, 295 (*n* = 48.8%) had previously experienced that the antimicrobials they were using on their farm were not working properly.

### 2.3. Knowledge of Antimicrobial Use and Resistance among Poultry Farmers

Most of the poultry farmers (87.8%; 531/605) used antibiotics and antimicrobials for various purposes on their birds. Only 49.8% (*n* = 301/605) of them were aware of antimicrobial resistance (AMR). Almost three-fourths of the poultry farmers (73.4%; 444/605) thought that antimicrobials or antibiotics had some side effects, and more than half of them (55.7%; *n* = 337/605) had knowledge about antimicrobial residue. Approximately 60% (*n* = 361/605) of the farmers believed that antimicrobials used in poultry had the potential to reach the human body through the intake of animal products and 55.7% (*n* = 337/605) of them knew that the misuse of antimicrobials could affect human and environmental health. The mean knowledge scores of backyard, broiler, and layer farmers were 5.74 ± 3.62, 8.34 ± 2.83, and 8.97 ± 2.66, respectively. There were significant differences in the knowledge of AMR among the farmers rearing backyard, broiler, and layer chickens (*p* < 0.05) (Table 1).

### 2.4. Effect of Sociodemographic Factors on the Knowledge Level of Antimicrobial Use and Resistance

Regarding knowledge scores, 49.4% (*n* = 299/605) of the total population achieved at least 75% of total knowledge score (good knowledge). The multivariable logistic regression analysis revealed that the age, gender, level of education, district, and types of birds were significantly associated with higher knowledge scores. Therefore, farmers aged 45 and above were 47% less likely (OR: 0.53; 95% CI: 0.28–0.99) to have good knowledge compared to the farmers within the age group 18–29. Likewise, we found increased odds of having good knowledge in males compared to females (OR: 2.26; 95% CI: 1.47–3.47). Furthermore, the odds of having good knowledge enhanced as the level of education increased and participants with tertiary education (above class 12) had 5.09 (95% CI: 1.96–13.22) times higher odds when compared with the farmers with no formal education. Participants from the Kailali districts had 6.96 (95% CI: 2.90–16.70) times significantly higher odds of having good knowledge compared to participants from the Syangja district. The farmers’ rearing layers had significantly higher odds compared to backyard poultry farmers (OR: 2.5; 95% CI: 1.39–4.47) (Table 2).

### 2.5. Attitude toward Antimicrobial Use and Resistance

Most poultry farmers (91.6%; *n* = 554/605) agreed that seeking the advice of a veterinarian is necessary before using antimicrobials. Nearly three-quarters of the study population accepted that good hygiene (79.2%; *n* = 479/605) and biosecurity (73.6%; *n* = 445/605) can reduce the problem of AMR. More than half of the participants admitted that reducing the use of antimicrobials or antibiotics can promote human health (67.3%; *n* = 407/605) and environmental health (57.4%; *n* = 347/605), respectively. The mean attitude scores of farmers rearing backyard, broiler, and layers are 4.3 ± 2.79, 6.16 ± 1.92, and 6.71 ± 1.7, respectively. Like the knowledge, each attitude level varied significantly among the farmers rearing the different kinds of birds (*p* < 0.05) (Table 3).

### 2.6. Effect of Sociodemographic Factors on Attitude towards Prudent Antimicrobial Usage

A total of 62.8% (*n* = 380/605) of the participants in our study had an attitude score of at least 75% (good attitude). In the multivariable model, all the predictors except age and experience were significantly associated with the attitude score. The male participants had higher odds of having a good attitude score than the female participants (OR: 1.62; 95% CI: 1.02–2.56). Participants with higher secondary education (class 11–12) had 3.10 (95% CI: 1.42–6.78) and participants with tertiary education (above class 12) had 7.85 (95% CI: 2.67–23.08) times higher odds of having a good attitude score when compared to those with no formal education. Likewise, farmers from the Kailali district had 2.51 (95% CI: 0.88–7.20) times higher odds of having a good attitude when compared with farmers from the Syangja district. Furthermore, layer farmers had 4.02 (95% CI: 2.12–7.64) times higher odds of having a good attitude score compared to farmers rearing backyard chickens (Table 4).

### 2.7. The Practice of Antimicrobial Use

Our research findings revealed that only half of the study population followed appropriate practices either by calling veterinarians (16.4%; *n* = 99/605) or taking their sick birds to hospitals or clinics (33.9%; *n* = 205/605). The remaining participants either practiced self-treatment (11.2%; 68/605), called para-veterinarians (19.8%; 120/605), or went to a local drug seller (18.7%; *n* = 113/605) for treatment. One-fifth (20.3%; 123/605) of the study population maintained the withdrawal period before selling or consuming the poultry products. A large percentage of the poultry farmers (84.1%; 509/605) mentioned that they referred to the guidelines before administering the antimicrobials. Almost 71% (*n* = 429/605) of the respondents obtained prescriptions from veterinarians before buying the antimicrobials. A total of 47.6% (*n* = 288/605)) of the farmers used antimicrobials exclusively for treatment, and almost 29% (*n* = 175/605) of the population used antimicrobials to increase production. Only 26% (*n* = 157/605) of the farmers in our study visited the laboratory to perform an antimicrobial susceptibility test (AST) before using antibiotics on their farms. Approximately 15% (*n* = 89/605) of the farmers used antimicrobials for the duration prescribed by the veterinarian, and 30.4% (*n* = 184/605) of them used antibiotics for 5–7 days. Unfortunately, 32.4% (*n* = 196/605) of the population stopped using the antimicrobials as soon as the disease symptoms subsided. Almost 45% (*n* = 270/605) of the study population kept records of the antimicrobials they used in the past, while the remaining did not. The mean practice scores of farmers raising backyard, broiler, and layer chickens were 5.12 ± 3.42, 8.13 ± 2.83, and 9.19 ± 3.02, respectively. There were also significant differences in the responses of poultry farmers who reared backyard, broiler, and poultry chickens in Nepal (*p* < 0.05) (Table 5).

### 2.8. Effect of Sociodemographic Factors on Practices Associated with Prudent Antimicrobial Use

Unlike the knowledge and attitude score, only 12.7% (*n* = 77/605) of the poultry farmers had at least 75% of the practice score (good practice) (Table 6). In the multivariable logistic model, male participants had higher odds of having good practices compared to female participants (OR: 3.36; 95% CI: 1.38–8.20). Similarly, farmers from the Kathmandu district had 16.56 (95% CI: 1.38–31.16) times higher odds of having good practice scores when compared to poultry farmers of the Syangja district. Likewise, participants rearing layer chickens had increased odds of having good practices when compared to participants rearing backyard chickens.

### 2.9. Correlation between Knowledge, Attitude, and Practice

**Figure 2:**
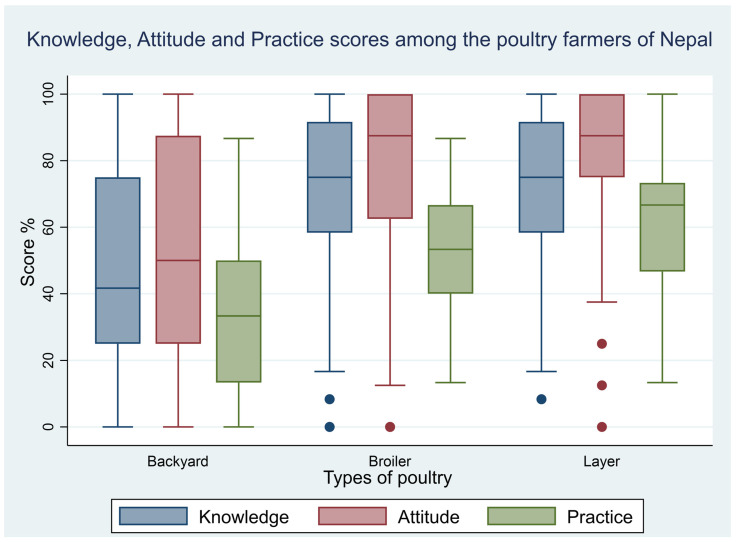
Box plot of KAP scores among different poultry farmers. For the above plot, KAP scores were standardized (divided by total score) and were transformed into percentages.

The Spearman’s rank correlation test revealed a positive correlation coefficient between the KAP scores (Figure 2). The correlation between knowledge and attitude scores was 0.63 (95% CI: 0.58–0.69). Similarly, the correlation between knowledge and practice score was 0.55 (95% CI: 0.49–0.61). The lowest correlation coefficient was between attitude and practice with a value of 0.44 (95% CI: 0.37–0.50) (Table 7). The correlation between knowledge and attitude as well as between knowledge and practice was moderately positive and the correlation between attitude and practice was marginally positive [19].

## 3. Discussion

In the global efforts to control antimicrobial resistance (AMR), a lack of sufficient strategies to address the indiscriminate use of antimicrobials and inadequate application of the available laws and policies are the major constraints in Nepal. In the context of Nepal, relatively modest investments in intervention strategies to fight AMR are immediately required [20]. In Kathmandu (the capital city of Nepal), 90% of poultry farms (Broiler) use antibiotics either for treatment or prophylaxis [13,21].

The knowledge level of participants regarding different issues of AMU and AMR in our study was relatively high. Similar findings were reported in a study conducted in Bangladesh [22]. However, the level of knowledge was relatively low in studies conducted in Cameroon and India [23,24]. The level of knowledge of participants in our study could be higher as a higher proportion of them were from some of the developed districts of Nepal including Kathmandu. The majority of the population in our study was aware of antibiotics and their use. However, only half of the participants knew about AMR and ways to reduce it. The result suggests the presence of a knowledge gap between prudent AMU and AMR. Participants aged 45 and above had significantly lower knowledge levels compared to farmers aged 18–29, which is similar to the study conducted in Bangladesh [22]. This may be due to the increasing literacy rate and awareness of AMR from social media. Youths have more access to social media and are more flexible in accepting and following new issues like AMR. Additionally, the male population had significantly higher levels of knowledge compared to females. In Nepal, major business entrepreneurship is male-dominated, and compared to females, a high proportion of males are in decision-making positions, which could have influenced the knowledge level across the genders [25,26]. Furthermore, the low participation of women in technical and vocational training, compared to males, may have influenced the lower KAP values in females [27] Again, lesser knowledge of the female population could be a major challenge to reducing AMR in Nepal as, unlike decision-making, the majority of women are involved in day-to-day activities related to livestock (including poultry) like feeding and cleaning [28,29]. A lower participation (30%; *n* = 179) of women was observed in our study, which depicts the situation of agriculture entrepreneurship in Nepal. In the current study, the knowledge level varied according to the educational level, and participants with higher education had significantly higher odds of having better knowledge status. Similar results were obtained in the studies in Bangladesh [22], Romania [30], Hong Kong [31], Turkey [32], Vietnam [33], Germany [34], and five African countries [35]. This suggests that education plays a vital role in making people aware of the concepts of AMU and AMR. Our study showed that farmers from the Kailali district had significantly higher levels of knowledge compared to others. This could be due to the rapid growth of the poultry sector in Kailali over the last decade, with a large number of big poultry farms, hatcheries, and feed companies [36].

Like knowledge, the study population had a good attitude toward AMR and AMU. The average score of the participants was 72.5%, which again could be attributed to the sampling location (major poultry hubs like Chitwan, Kailali, and Kathmandu). The majority of the participants agree on the fact that advice from the veterinarian is necessary before the use of antimicrobials on their farms. Similarly, most of them believe that good hygiene, biosecurity, vaccination, education, and awareness can play a vital role in reducing the problem of AMR. This is overwhelming, but we must keep in mind that, still, a considerable number of people do not agree with these facts. Addressing the problem of AMR is never an individual task and requires a collaborative effort from all the stakeholders [37,38,39]. Again, the level of attitude also varied among the various demographic variables. Males again had better attitudes when compared to females and participants of the Kailali districts had better attitudes toward AMU when compared with participants of other districts. The attitude levels were very similar to the knowledge level as they had a significant positive correlation between them. This association is also attributed to the fact that knowledge level could have influenced the level of attitudes in participants, as similar results were obtained in previous studies too [22,40]. However, unlike knowledge levels, it is rather surprising to see the lower level of attitude among farmers of Kathmandu and Chitwan compared to Syangja. This provides us with knowledge of the fact that, despite having good knowledge, people of so-called developed districts do not necessarily show positive attitudes. This is where strong federal, provincial, and local level rules and policies against AMR should act.

In our study, a clear transfer of knowledge and attitude into practice was not observed. The average practice score was 50.6% compared to 65.1% and 72.5% of knowledge and attitude scores, respectively. In this study, 11.24% of the respondents were involved in self-treatment and 29.09% did not obtain prescriptions from veterinarians before buying the antimicrobials. These practices hold significant public health risks and promote the development of antimicrobial resistance [2,41]. Additionally, only 26% of the respondents visited veterinary laboratories to perform antimicrobial susceptibility tests (ASTs) before using antimicrobials in their farms. As several antimicrobials are resistant to different types of pathogens, the use of antimicrobials without performing ASTs can elongate the duration of treatment and on multiple occasions could force the farmer to try multiple antimicrobials [21,42,43]. In this study, only 20.33% of the study population maintained the withdrawal period, which is less than half the value reported by a recent study held in the Chitwan district of Nepal [44]. Furthermore, almost half of the study population either used the antimicrobials for a few days or stopped using them as soon as the symptoms subsided. The inappropriate use of antimicrobials is always a leading factor in the development of AMR [45,46,47]. Despite the focus of the Government of Nepal on ensuring no antibiotics are found in feed supplements, almost half of the study population used antimicrobials primarily either for growth or for both growth and treatment [2,44]. Just like for knowledge and attitude, male participants from the Kailali district raising layer poultry had the highest practice level compared to others. However, education was statistically non-significant with the practice score. This is a matter of surprise as the previous studies showed improved practice levels with higher educational levels of their participants [22,48]. This could be attributed to low levels of practice in our study as well as provide an important insight into the fact that education can influence knowledge and attitude but not necessarily practice if other factors like regulations and awareness do not act in place.

In the current study, poultry farmers used 11 classes of antimicrobials. Out of them, four classes of antimicrobials, namely macrolides, cephalosporins (first and third generation), and polymyxins, are the highest priority as they are critically important antimicrobials in human medicine [49].

The World Health Organization suggests not to use these highest priority critically important antimicrobials for treatment and control of disease in food-producing animals [50]. The easy availability and over-the-counter (OTC) sale of these antimicrobials in the country makes it more difficult to stop their haphazard use. A recent study conducted in the Kathmandu Valley of Nepal had similar findings [21]. Additionally, nearly half of the population in our study revealed that they experienced treatment failure with some of the antimicrobials in their farms. This is a very concerning fact and reflects the situation of AMR in Nepal.

Overall, the KAP level of participants in this study was more or less similar to previous studies [24,44,51]. The current situation of understanding and using antimicrobials in Nepal should be upgraded to ensure a safe direction toward the use of antimicrobials and prevent AMR. Antimicrobial resistance carries a significant public health risk and can significantly hinder the treatment system in both humans and animals. Additionally, low- and middle-income countries (LMICs) like Nepal are the epicenter of increasing public health threats [39]. As revealed by our study, still a considerable part of the population does not know that vaccination and biosecurity can decrease the burden of antimicrobial use and hinder the development of AMR. Previous studies have highlighted the increased risks of AMR in farms with poor biosecurity and the role of vaccines in reducing the occurrence of infectious diseases and antimicrobial use [52,53,54,55]. Furthermore, the technical knowledge regarding antimicrobial use and its resistance should be enhanced among female farmers of Nepal. A mutual decision-making environment with the involvement of females in the business discussion may be essential to reduce the disparity of KAP levels among genders in Nepal.

Hence, regular awareness programs involving all the related stakeholders through multiple streams and the development of a new framework to fight AMR are crucial in Nepal. Despite the commencement of laboratory surveillance of AMR in 1999, there is a limited number of comprehensive studies on AMR in Nepal [56]. In 2014, the Alliance for the Prudent Use of Antibiotics (APUA) drafted the National Antibiotics Treatment Guidelines for Nepal, which was approved by the Ministry of Health and Population (MOHP) [57]. The guidelines were overwhelming but primarily focused on human health. As the animal sector in Nepal also contributes significantly to the development of AMR, the Government of Nepal should work in a One Health framework during the planning and drafting of the guidelines and policies. In 2016, the Government of Nepal launched the National Antimicrobial Resistance Containment Action Plan to protect Nepalese people from related risks of AMR [56]. However, for more than 6 years, very little has been achieved [58]. In the middle of all these dilemmas and challenges, Nepal should force a way out to join the battle against AMR by the effective application of the available policies and by developing alternative approaches to address AMR in a One Health framework.

Like any research, this study has certain limitations that should be taken into consideration. Firstly, the data were collected through personal interviews, which introduces the possibility that some farmers may have provided socially desirable answers, potentially affecting the accuracy of the responses. Secondly, the snowball sampling method employed in this survey could have influenced the results, as it relies on participants referring others who may share similar characteristics or opinions. This could introduce bias into the sample. Thirdly, this study did not explore the difference in KAP levels in rural, urban, and peri-urban areas of the study area, which could potentially have impacted the scores. However, we suggest that future researchers include this demographic variable in their study. Finally, it is important to note that the participants in this study were primarily from the major poultry areas of Nepal, such as Chitwan, Kathmandu, and Kailali. Therefore, the knowledge, attitude, and practice (KAP) levels observed in this study may not necessarily reflect the overall KAP levels of the entire country.

## 4. Materials and Methods

### 4.1. Study Area and Population

**Figure 3:**
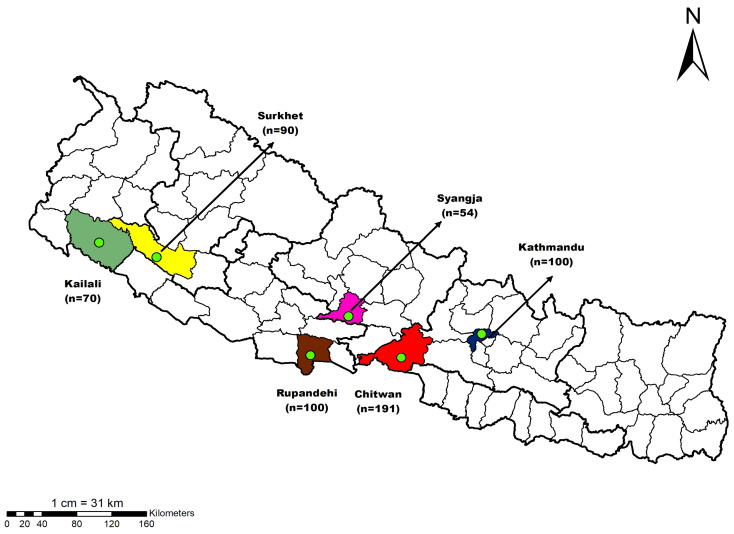
Map of Nepal showing districts from which the poultry farmers were recruited.

This study was conducted in 6 districts of Nepal: Kailali from Sudurpashim Province, Surkhet from Karnali Province, Rupandehi from Lumbini Province, Syangja from Gandaki Province, Chitwan, and Kathmandu from Bagmati Province (Figure 3). In Nepal, there are 29.19 million people, and among them, around 55 thousand engage in poultry farming, with chickens being produced commercially in 64 of the country’s 77 districts [59]. In the most recent report of the “Agriculture and Livestock Diary Report No. 2079” published on 17 April 2022, there are 73,418,077 hens and 427,226 ducks in Nepal and among them, 11,374,011 laying hens and 220,532 laying ducks [11]. Nepal produced 226,959 metric tons of chicken meat, 442 metric tons of duck meat, and around 1.47 billion chicken eggs and 17.9 million duck eggs in 2020/21. The study population was poultry farmers who were aged > 18 years, actively engaged in poultry production, and/or involved in the decision pattern of antimicrobial administration.

### 4.2. Study Design and Sample Size

To answer our research questions, we conducted a cross-sectional survey among poultry farmers from 6 districts of Nepal between 1 May 2022 and 15 June 2022. The sample size was calculated based on the assumption that 50% of poultry farmers were aware of AMR and prudently used antibiotics, a 95% degree of confidence, and a 5% level of error. A sample size of 384 was computed; furthermore, we added a 30% contingency and thus the minimum sample size for the study was 500. Snowballing (convenience) sampling was conducted in each district. A total of 605 farmers participated in this study: 70 from Kailali, 90 from Surkhet, 100 from Rupandehi, 54 from Syangja, 191 from Chitwan, and 100 from Kathmandu district.

### 4.3. Questionnaire Design and Data Collection

The questionnaire used in this study had four sections; 1. Demographics of poultry farmers, 2. Knowledge of antimicrobial use and resistance, 3. Attitude toward prudent antimicrobial usage and resistance, and 4. The practice of antimicrobial use. The obtained demographic information included age, gender, education, province, district, municipality, poultry type reared, and years of experience. The knowledge section had 12 different close-ended questions while the attitude section had 8 different close-ended questions. The practice section had 15 both closed and open-ended questions. A preliminary interview was conducted among 20 farmers in the Chitwan district to improve the quality of the questionnaire, but their data were not included in the analysis. Structured questionnaire interviews were conducted from May 2022 to June 2022. In each selected district of this province, data collection was conducted by trained veterinarians and veterinary students. Data were collected in both English and Nepali languages according to the preferences of the farmers. All poultry farmers regardless of their educational status and sex were included as participants in the study.

Additionally, to verify the internal consistency of the questionnaire, we assessed the value of Cronbach’s alpha. The Cronbach’s alpha for knowledge (K1–K12), attitude (A1–A8), and practice (P1–P15) were 0.826, 0.821, and 0.777, respectively. The value of Cronbach’s alpha suggested that the knowledge and attitude domain had good internal consistency, and the practice domain had acceptable internal consistency [59].

### 4.4. Data Analysis

The responses collected from the in-person interviews were fed into a spreadsheet using Microsoft Excel and the values were double-checked for completeness and accuracy. Data cleaning and analysis were performed using Stata (version 15.1). The assessment of knowledge, attitude, and practice was performed with a scoring system. The KAP scores of the participants were calculated as the sum of correct responses to each question. The correct response was scored 1 and the wrong response 0. The questionnaire contained the “Do not know” variable in the majority of the questions. Although it is a conservative approach, we decided to score the “Do not know” response as 0, as we believe the response is primarily from the respondents who are likely to have less knowledge [60]. The correct responses were added to obtain total knowledge, attitude, and practice scores, respectively. Each respondent could obtain a score of 0–12 for knowledge, 0–8 for attitude, and 0–15 for practice. To identify the normal distribution of the KAP score, the Shapiro–Wilk normality test was used. As the scores were not distributed normally, Spearman’s rank correlation was used to explore the correlation values between the KAP scores. A cut-off point of 75% was set to define the KAP score as “good” or “poor”, which means that scores above 8, 5, and 11 for knowledge, attitude, and practice were considered good. We employed a binary scoring system (categorizing as good or poor) to achieve simplicity, easy interpretation, and efficient resource allocation. This approach enables straightforward comparisons, strengthens the study’s conclusions, and has practical applications while preventing overfitting and enhancing generalizability.

A logistic regression analysis was performed to identify the predictors of good KAP status. Independent variables like age, gender, education level, district, types of poultry, and education were included as predictors. The continuous variable experience (in years) was converted into high and low using a median value to address the issue of linearity in the logistic model. The KAP levels of “good” vs.“poor” were the dependent variable in the mode. A univariate logistic regression analysis was performed between the independent variables and KAP levels and the variables having a significance of *p* < 0.2 were considered for the multivariate model. The multicollinearity between the independent variables was verified with variance inflation factor (VIF) using the “collin” command in Stata. The Hosmer–Lameshow test was used to predict the goodness of fit of the final KAP models.

## 5. Conclusions

Our findings showed good knowledge and attitude scores but a relatively low level of desirable practice scores, reflecting the presence of an implementation gap among poultry farmers. Additionally, our findings reveal that KAP scores among farmers rearing different species of birds were significantly different, and farmers rearing backyard chickens had lower mean KAP scores. Furthermore, respondents’ socio-demographic factors were significantly associated with good knowledge, attitude, and practices among poultry farmers. The over-the-counter sales of antimicrobials in Nepal should be regulated. Farmers should be encouraged to perform antimicrobial susceptibility tests and obtain prescriptions from registered veterinarians before using any antimicrobials. Overall, multi-sectoral collaborative efforts are necessary to promote the prudent use of antimicrobials among poultry farmers in Nepal.

## Figures and Tables

**Table 1:** Knowledge about antimicrobial use and resistance among different poultry farmers.

S.N.	Variable	Type of Birds *n* (%)			Total *n* (%)	*p*-Value
		Backyard	Broiler	Layer		
**1**	**Do You Use Antibiotics/Antimicrobials?**					
	No	28 (17.95)	37 (11.75)	9 (6.72)	74 (12.23)	0.013
	**Yes**	128 (82.05)	278 (88.25)	125 (93.28)	531 (87.77)	
**2**	**Do you know who has the authority to prescribe antibiotics?**					
	No	74 (47.44)	81 (25.71)	33 (24.63)	188 (31.07)	<0.001
	**Yes**	82 (52.56)	234 (74.29)	101 (75.37)	417 (68.93)	
**3**	**Do you think antimicrobials/antibiotics have some side effects?**					
	No	54 (34.62)	77 (24.44)	30 (22.39)	161 (26.61)	0.029
	**Yes**	102 (65.38)	238 (75.56)	104 (77.61)	444 (73.39)	
**4**	**Do you think the entire flock should be treated if one or a few birds display symptoms?**					
	No	62 (39.74)	90 (28.57)	37 (27.61)	189 (31.24)	0.028
	**Yes**	94 (60.26)	225 (71.43)	97 (72.39)	416 (68.76)	
**5**	**Do you know about antimicrobial residue?**					
	No	100 (64.10)	116 (36.83)	52 (38.81)	268 (44.30)	<0.001
	**Yes**	56 (35.90)	199 (63.17)	82 (61.19)	337 (55.70)	
**6**	**Do you know what antimicrobial resistance (AMR) is?**					
	No	99 (63.46)	150 (47.62)	55 (41.04)	304 (50.25)	<0.001
	**Yes**	57 (36.54)	165 (52.38)	79 (58.96)	301 (49.75)	
**7**	**Do you think antimicrobials used in poultry carry the potential to reach the human body through the intake of animal products?**					
	No	90 (57.69)	104 (33.02)	50 (37.31)	244 (40.33)	<0.001
	**Yes**	66 (42.31)	211 (66.98)	84 (62.69)	361 (59.67)	
**8**	**Can you reduce AMR development by avoiding the overuse of antimicrobials in animal production?**					
	No	110 (70.51)	138 (43.81)	43 (32.09)	291 (48.10)	<0.001
	**Yes**	46 (29.49)	177 (56.19)	91 (67.91)	314 (51.90)	
**9**	**Do you know that the unwise use of antimicrobials affects human and environmental health?**					
	No	97 (62.18)	123 (42.22)	38 (28.36)	268 (44.30)	<0.001
	**Yes**	59 (37.82)	182 (57.78)	96 (71.64)	337 (55.70)	
**10**	**Do you think that good biosecurity measures in farms can reduce the problem of AMR?**					
	No	80 (51.28)	59 (18.73)	22 (16.42)	161 (26.61)	<0.001
	**Yes**	76 (48.72)	256 (81.27)	112 (83.58)	444 (73.39)	
**11**	**Do you know that the routine vaccination of farm animals can reduce the AMR problem?**					
	No	102 (65.38)	114 (36.19)	22 (16.42)	238 (39.34)	<0.001
	**Yes**	54 (34.62)	201 (63.81)	112 (83.58)	367 (60.66)	
**12**	**Do you know that good hygiene practices on farms can reduce the problem of AMR?**					
	No	80 (51.28)	53 (16.83)	15 (11.19)	148 (24.46)	<0.001
	**Yes**	76 (48.72)	262 (83.17)	119 (88.81)	457 (75.54)	

All the *p*-values are derived from the chi-squared test. Responses in bold represent correct answers.

**Table 2:** Logistic regression analysis of the predictors of good knowledge of AMU and AMR.

Characteristics	Knowledge Level		OR (95% CI)	*p*-Value	Adjusted OR (95% CI)	*p*-Value
	Poor (%)	Good (%)				
**Age**						
30–44	150 (45.73)	178 (54.27)	0.99 (0.63–1.55)	0.001	1.05 (0.61–1.79)	0.008
45 and above	111 (62.36)	67 (37.64)	0.5 (0.31–0.83)		0.53 (0.28–0.99)	
18–29	45 (45.45)	54 (54.55)	Ref.		Ref.	
**Gender**						
Male	181 (42.49)	245 (57.51)	3.13 (2.16–4.55)	<0.001	2.26 (1.47–3.47)	0.0002
Female	125 (69.83)	54 (30.17)	Ref.		Ref.	
**Education**						
Primary education (1–5)	38 (60.32)	25 (39.68)	1.73 (0.80–3.71)	<0.001	1.79 (0.76–4.21)	0.0102
Lower secondary (6–8)	60 (61.86)	37 (38.14)	1.62 (0.80–3.28)		1.61 (0.73–3.55)	
Secondary (9–10)	86 (54.09)	73 (45.91)	2.23 (1.16–4.29)		1.86 (0.87–3.96)	
Higher Secondary (11–12)	67 (38.51)	107 (61.49)	4.19 (2.18–8.04)		2.83 (1.31–6.14)	
Tertiary education (>12)	13 (24.07)	41 (75.93)	8.28 (3.54–19.35)		5.09 (1.96–13.22)	
No formal education	42 (72.41)	16 (27.59)	Ref.	<0.001	Ref.	
**District**						
Surkhet	47 (52.22)	43 (47.78)	3.20 (1.49–6.87)	<0.001	3.66 (1.63–8.21)	<0.001
Chitwan	85 (44.50)	106 (55.50)	4.36 (2.16–8.81)		2.93 (1.39–6.21)	
Kathmandu	40 (40)	60 (60)	5.25 (2.46–11.18)		5.80 (2.53–13.29)	
Kailali	21 (30)	49 (70)	8.16 (3.60–18.55)		6.96 (2.90–16.70)	
Rupandehi	71 (71)	29 (29)	1.43 (0.66–3.10)		1.79 (0.75–4.30)	
Syangja	42 (77.78)	12 (22.22)	Ref.		Ref.	
**Birds type**						
Broiler	150 (47.62)	165 (52.38)	2.48 (1.65–3.71)		1.70 (1.06–2.73)	0.0082
Layer	48 (35.82)	86 (64.18)	4.03 (2.47–6.58)		2.50 (1.39–4.47)	
Backyard	108 (69.23)	48 (30.77)	Ref.	<0.001	Ref.	
**Experience**						
4 years and above	149 (45.43)	179 (54.57)	1.57 (1.14–2.17)		1.33 (0.89–1.98)	0.1651
0–3 years	157 (56.68)	120 (43.32)	Ref.	0.006	Ref.	

**Table 3:** Attitude toward prudent antimicrobial usage and resistance among different poultry farmers in Nepal (*n* = 605).

S.N.	Variable	Type of Birds *n* (%)			Total *n* (%)	*p*-Value
		Backyard	Broiler	Layer		
**1**	**Is seeking advice from a veterinarian necessary before using antimicrobials?**					
	Disagree	33 (21.15)	15 (4.76)	3 (2.24)	51 (8.43)	<0.001
	**Agree**	123 (78.85)	300 (95.24)	131 (97.76)	554 (91.57)	
**2**	**Good hygiene can reduce the problem of AMR.**					
	Disagree	71 (45.51)	44 (13.97)	11 (8.21)	126 (20.83)	<0.001
	**Agree**	85 (54.49)	271 (86.03)	123 (91.79)	479 (79.17)	
**3**	**Biosecurity can reduce the problem of AMR.**					
	Disagree	79 (50.64)	68 (21.59)	13 (9.70)	160 (26.45)	<0.001
	**Agree**	77 (49.36)	247 (78.41)	121 (90.30)	445 (73.55)	
**4**	**Vaccination can reduce the problem of AMR.**					
	Disagree	93 (59.62)	88 (27.94)	22 (16.42)	203 (33.55)	<0.001
	**Agree**	63 (40.38)	227 (72.06)	112 (83.58)	402 (66.45)	
**5**	**Antimicrobial use regulation can decrease the irrational use of antimicrobials in animals.**					
	Disagree	81 (51.92)	85 (26.98)	40 (29.85)	206 (34.05)	<0.001
	**Agree**	75 (48.08)	230 (73.02)	94 (70.15)	399 (65.95)	
**6**	**Education and public awareness reduce the problem of AMR.**					
	Disagree	62 (39.74)	52 (16.51)	14 (10.45)	128 (21.16)	<0.001
	**Agree**	94 (60.26)	263 (83.49)	120 (89.55)	477 (78.84)	
**7**	**Reducing the use of antimicrobials or antibiotics in livestock can promote human health.**					
	Disagree	73 (46.79)	97 (30.79)	28 (20.90)	198 (32.73)	<0.001
	**Agree**	83 (53.21)	218 (69.21)	106 (79.10)	407 (67.27)	
**8**	**Reducing the use of antimicrobials or antibiotics in livestock can promote environmental health.**					
	Disagree	85 (54.49)	131 (41.59)	42 (31.34)	258 (42.64)	<0.001
	**Agree**	75 (45.51)	184 (58.41)	92 (68.66)	347 (57.36)	

All the *p*-values are derived from the chi-squared test. Responses in bold represent correct answers.

**Table 4:** Logistic regression analysis of the predictors of good attitude towards prudent AMU.

			Univariable LR		Multivariable LR	
Characteristics	Attitude Level		OR (95% CI)	*p*-Value	Adjusted OR (95% CI)	*p*-Value
	Poor (%)	Good (%)				
**Age**						
30–44	118 (35.98)	210 (64.02)	0.63 (0.38–1.05)	0.006	0.7 (0.38–1.27)	0.222
45 and above	81 (45.51)	97 (54.49)	0.43 (0.25–0.73)		0.54 (0.27–1.09)	
18–29	26 (26.26)	73 (73.74)	Ref.		Ref.	
**Gender**						
Male	140 (32.86)	286 (67.14)	1.85 (1.29–2.64)	0.001	1.62 (1.02–2.56)	0.039
Female	85 (47.49)	94 (52.51)	Ref.		Ref.	
**Education**						
Primary education (1–5)	31 (49.21)	32 (50.79)	1.69 (0.82–3.49)	<0.000	2.12 (0.89–5.09)	0.005
Lower secondary (6–8)	41 (42.27)	56 (57.73)	2.24 (1.15–4.35)		1.94 (0.89–4.23)	
Secondary (9–10)	64 (40.25)	95 (59.75)	2.43 (1.31–4.51)		2.01 (0.94–4.27)	
Higher secondary (11–12)	45 (25.86)	129 (74.14)	4.69 (2.5–8.81)		3.10 (1.42–6.78)	
Tertiary education (>12)	8 (14.81)	46 (85.19)	9.41 (3.75–23.59)		7.85 (2.67–23.08)	
No formal education	36 (62.07)	22 (37.93)	Ref.		Ref.	
**District**						
Surkhet	28 (31.11)	62 (68.89)	0.70 (0.33–1.51)	<0.000	0.76 (0.34–1.72)	<0.000
Chitwan	77 (40.31)	114 (59.69)	0.47 (0.24–0.93)		0.27 (0.13–0.57)	
Kathmandu	27 (27.00)	73 (73.00)	0.86 (0.40–1.84)		0.93 (0.40–2.18)	
Kailali	7 (10.00)	63 (90.00)	2.85 (1.05–7.75)		2.51 (0.88–7.20)	
Rupandehi	73 (73.00)	27 (27.00)	0.12 (0.05–0.25)		0.11 (0.05–0.27)	
Syangja	13 (24.07)	41 (75.93)	Ref.		Ref.	
**Birds type**						
Broiler	107 (33.97)	208 (66.03)	2.72 (1.83–4.04)	<0.000	1.36 (0.833–2.32)	<0.000
Layer	27 (20.15)	107 (79.85)	5.55 (3.27–9.41)		4.02 (2.12–7.64)	
Backyard	91 (58.33)	65 (41.67)	Ref.		Ref.	
**Experience**						
4 years and above	106 (32.32)	222 (67.68)	1.58 (1.32–2.20)	0.007	1.40 (0.91–2.15)	0.128
0–3 years	119 (42.96)	158 (57.04)	Ref.		Ref.	

**Table 5:** The practice of antimicrobial use and resistance among different poultry farmers in Nepal (*n* = 605).

S.N.	Variable	Type of Birds *n* (%)			Total *n* (%)	*p*-Value
		Backyard	Broiler	Layer		
**1**	**What do you mostly do when your birds get sick?**					
	Self-treatment	31 (19.87)	30 (9.52)	7 (5.22)	68 (11.24)	<0.001
	Call para-veterinarian	45 (28.85)	57 (18.10)	18 (13.43)	120 (19.83)	
	Take to veterinary hospitals/clinics	42 (26.92)	114 (36.19)	49 (36.57)	205 (33.88)	
	Call veterinarians	12 (7.69)	61 (19.37)	26 (19.40)	99 (16.36)	
	Go to a local drug seller	26 (16.67)	53 (16.83)	34 (25.37)	113 (18.68)	
**2**	**Do you maintain the withdrawal period before consuming or selling poultry products?**					
	No	133 (85.26)	263 (83.49)	86 (64.18)	482 (79.67)	<0.001
	**Yes**	23 (14.74)	52 (16.51)	48 (35.82)	123 (20.33)	
**3**	**Do you refer to guidelines or calculate the dose while administering the antimicrobials?**					
	No	57 (36.54)	27 (8.57)	12 (8.96)	96 (15.87)	<0.001
	**Yes**	99 (63.46)	288 (91.43)	122 (91.04)	509 (84.13)	
**4**	**Do you obtain prescriptions from veterinarians before you buy drugs?**					
	No	81 (51.92)	78 (24.76)	17 (12.69)	176 (29.09)	<0.001
	**Yes**	75 (48.08)	237 (75.24)	117 (87.31)	429 (70.91)	
**5**	**Do you use separate boots or slippers inside the shed?**					
	No	87 (55.77)	49 (15.56)	21 (15.67)	157 (25.95)	<0.001
	**Yes**	69 (44.23)	266 (84.44)	113 (84.33)	448 (74.05)	
**6**	**For what purpose do you use antimicrobials most?**					
	Treatment	64 (41.03)	158 (50.16)	66 (49.25)	288 (47.60)	<0.001 *
	To increase production	52 (33.33)	86 (27.30)	37 (27.61)	175 (28.93)	
	Both for treatment and production	23 (14.74)	71 (22.54)	29 (21.64)	123 (20.33)	
	**I do not use**	17 (10.90)	0 (0)	2 (1.49)	19 (3.14)	
**7**	**Do you visit veterinary labs to perform antimicrobial susceptibility tests before using antibiotics on your farm?**					
	No	123 (78.85)	238 (75.56)	87 (64.93)	448 (74.05)	0.018
	**Yes**	33 (21.15)	77 (24.44)	47 (35.07)	157 (25.95)	
**8**	**Do you conduct routine vaccinations in your livestock as per the schedule?**					
	No	104 (66.67)	75 (23.81)	23 (17.16)	202 (33.39)	<0.001
	**Yes**	52 (33.33)	240 (76.19)	111 (82.84)	403 (66.61)	
**9**	**Do you practice spraying and wheel-dipping at your farm?**					
	No	115 (73.72)	113 (35.87)	42 (31.34)	270 (44.63)	<0.001
	**Yes**	41 (26.28)	202 (64.13)	92 (68.66)	335 (55.37)	
**10**	**When you start using antimicrobials, how long do you continue it?**					
	1–2 days	13 (8.33)	10 (3.17)	1 (0.75)	24 (3.97)	<0.001
	3–4 days	30 (19.23)	64 (20.32)	18 (13.43)	112 (18.51)	
	**5–7 days**	20 (12.82)	88 (27.94)	76 (56.72)	184 (30.41)	
	I stop medications as soon as the symptoms subside	71 (45.51)	96 (30.48)	29 (21.64)	196 (32.40)	
	**As prescribed by the veterinarian**	22 (14.10)	57 (18.10)	10 (7.46)	89 (14.71)	
**11**	**Do you keep a record of antimicrobials you used earlier on the farm?**					
	No	110 (70.51)	167 (53.02)	58 (43.28)	335 (55.37)	<0.001
	**Yes**	46 (29.49)	148 (46.98)	76 (56.72)	270 (44.63)	
**12**	**Do you have (use) a footbath before letting others enter and leave your farm?**					
	No	124 (79.49)	172 (54.60)	77 (57.46)	373 (61.65)	<0.001
	**Yes**	32 (20.51)	143 (45.40)	57 (42.54)	232 (38.35)	
**13**	**Do you regularly fumigate your farm?**					
	No	131 (83.97)	254 (80.63)	79 (58.96)	464 (76.69)	<0.001
	**Yes**	25 (16.03)	61 (19.37)	55 (41.04)	141 (23.31)	
**14**	**Do you have fencing around your farm?**					
	No	49 (31.41)	70 (22.22)	17 (12.69)	136 (22.48)	
	**Yes**	107 (68.59)	245 (77.78)	117 (87.31)	469 (77.52)	0.001
**15**	**Do you use an apron or separate cloth inside the shed?**					
	No	135 (86.54)	201 (63.81)	87 (64.93)	423 (69.92)	<0.001
	**Yes**	21 (13.46)	114 (36.19)	47 (35.07)	182 (30.08)	

All the *p*-values are derived from the chi-squared test except those in asterisk which are derived from Fisher’s exact test. Responses in bold represent correct answers.

**Table 6:** Logistic regression analysis of predictors of good practices toward prudent AMU and AMR.

			Univariable LR		Multivariable LR	
Characteristics	Practice Level		OR (95% CI)	*p*-Value	Adjusted OR (95% CI)	*p*-Value
	Poor (%)	Good (%)				
**Age**						
30–44	286 (87.20)	42 (12.80)	1.31 (0.63–2.71)	0.641		
45 and above	153 (85.96)	25 (14.04)	1.45 (0.67–3.17)			
18–29	89 (89.90)	10 (10.10)	Ref.			
**Gender**						
Male	356 (83.57)	70 (16.43)	4.83 (2.18–10.73)	<0.000	3.36 (1.38–8.20)	0.008
Female	172 (96.09)	7 (3.91)	Ref.		Ref.	
**Education**						
Primary education (1–5)	60 (95.24)	3 (4.76)	0.53 (0.12–2.32)	0.186	0.41 (0.09–2.01)	0.17
Lower secondary (6–8)	86 (88.66)	11 (11.34)	1.36 (0.45–4.12)		1.93 (0.58–6.46)	
Secondary (9–10)	136 (85.53)	23 (14.47)	1.79 (0.65–4.96)		1.87 (0.59–5.93)	
Higher secondary (11–12)	150 (86.21)	24 (13.79)	1.70 (0.62–4.67)		1.09 (0.34–3.53)	
Tertiary education (>12)	43 (79.63)	11 (20.37)	2.71 (0.88–8.40)		1.92 (0.54–6.86)	
No formal education	53 (91.38)	5 (8.62)	Ref.		Ref.	
**District**						
Surkhet	88 (97.78)	2 (2.22)	0.59 (0.08–4.32)	<0.000	0.63 (0.08–4.79)	<0.000
Chitwan	167 (87.43)	24 (12.57)	3.74 (0.85–16.34)		1.83 (0.39–8.58)	
Kathmandu	79 (79.00)	21 (21.00)	6.91 (1.55–30.73)		6.56 (0.38–31.16)	
Kailali	48 (68.57)	22 (31.43)	11.92 (2.66–53.40)		13.30 (2.80–63.33)	
Rupandehi	94 (94.00)	6 (6.00)	1.66 (0.32–8.52)		1.90 (0.34–10.63)	
Syangja	52 (96.30)	2 (3.70)	Ref.		Ref.	
**Birds type**						
Broiler	275 (87.30)	40 (12.70)	3.1 (1.35–7.08))	<0.000	1.94 (0.79–4.78)	0.003
Layer	104 (77.61)	30 (22.39)	6.14 (2.6–14.51)		4.63 (1.75–12.25)	
Backyard	149 (95.51)	7 (4.49)	Ref.		Ref.	
**Experience**						
4 years and above	275 (83.84)	53 (16.16)	2.03 (1.22–3.39)	0.006	1.61 (0.89–2.90)	0.114
0–3 years	253 (91.34)	24 (8.66)	Ref.		Ref.	

95% CI—95% Confidence interval; LR—logistic regression.

**Table 7:** Spearman rank correlation coefficients between the KAP scores.

Variables	Spearman Rank Correlation Coefficients (95% CI)	*p*-Value
Knowledge–attitude	0.63 (0.58–0.69)	<0.0001
Knowledge–practice	0.55 (0.49–0.61)	<0.0001
Attitude–practice	0.44 (0.37–0.50)	<0.000

The confidence interval was generated by bootstrap code in STATA with 1000 replications.

## Data Availability

Data will be available upon reasonable request.

## Supplementary Materials

The following supporting information can be downloaded at: https://www.mdpi.com/article/10.3390/antibiotics12091369/s1, Figure S1. Percentage of antibiotics classes used by poultry farmers in Nepal (*n* = 605).
