# Supplementary material for: Knowledge, Attitude, and Practice of Antibiotic Use and Resistance among Poultry Farmers in Nepal
Source: Antibiotics (Basel). 2023 Aug 25;12(9):1369. doi: 10.3390/antibiotics12091369 (PMC10525118; doi:10.3390/antibiotics12091369)
Supplement: Supplementary file 1 [file antibiotics-12-01369-s001.zip › antibiotics-2533152-supplementary.pdf]

Supplementary Files

Figure S1.

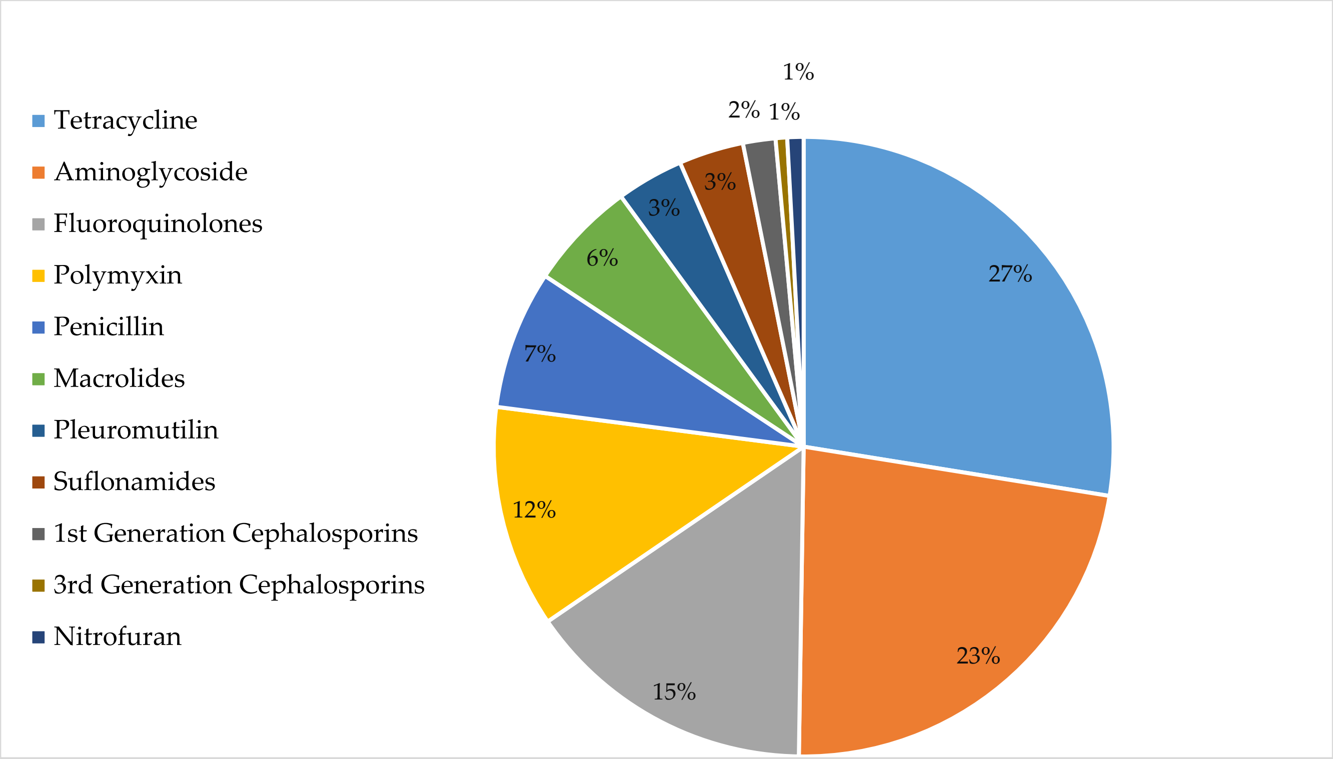

Figure S1. Percentage of antibiotics classes used by poultry farmers in Nepal (n = 605).
